# Elevated Interleukin-6 Is Associated with Successful Weight Loss 3 Months Postlaparoscopic Sleeve Gastrectomy

**DOI:** 10.1007/s11695-024-07468-y

**Published:** 2024-08-24

**Authors:** Marietta Bracha, Alina Jaroch, Adrian Falkowski, Beata Zwierko, Magdalena Szwed, Maciej Michalik, Alina Borkowska, Krzysztof Szwed, Mariusz Kozakiewicz

**Affiliations:** 1 Department of Geriatrics, Division of Biochemistry and Biogerontology, Faculty of Health Sciences, Collegium Medicum in Bydgoszcz, Nicolaus Copernicus University in Torun, 85626 Bydgoszcz, Poland; 2 Department of Clinical Neuropsychology, Faculty of Health Sciences, Collegium Medicum in Bydgoszcz, Nicolaus Copernicus University in Torun, 85094 Bydgoszcz, Poland; 3 Department of Probability Theory and Stochastic Analysis, Faculty of Mathematics and Computer Science, Nicolaus Copernicus University in Torun, 87100 Torun, Poland; 4 Department of General and Minimally Invasive Surgery, Faculty of Medicine, Collegium Medicum in Bydgoszcz, Nicolaus Copernicus University in Torun, 85168 Bydgoszcz, Poland; 5 The Mazovian Academy in Plock, 09402 Plock, Poland

**Keywords:** Bariatric surgery, Interleukin-6, Interleukin-10, Tumor necrosis factor-α, Body measures

## Abstract

**Purpose:**

Bariatric surgery poses an ever-increasing importance in the effective and long-lasting treatment of obesity, a condition strongly associated with inflammation and increased risk of other diseases and health problems. In obesity-related inflammation, maintaining a balance between pro-inflammatory and anti-inflammatory cytokines is crucial. In this study, we examined early effects of laparoscopic sleeve gastrectomy (LSG) on inflammatory and anti-inflammatory cytokines in obese patients, and assessed their effect on postoperative weight loss.

**Materials and Methods:**

This prospective cohort study was conducted from September 2022 till June 2023. Fifty obese adults were enrolled for LSG. All patients underwent assessments of body measurements, as well as levels of interleukin-6 (IL-6), interleukin-10 (IL-10), and TNF-alpha at baseline and 3 months postsurgery. We developed a decision tree model to predict the success of weight loss.

**Results:**

At 3 months postsurgery, patients lost 18.9 ± 6.9 kg of excess body weight. A significant decrease was observed for IL-10 (*p* < 0.0001), simultaneously with a significant increase in IL-6 (*p* < 0.0001). We found that high IL-6 (> 1.169 pg/mL) levels could contribute to an effective weight loss among patients with a baseline BMI less than 47.46 kg/m^2^.

**Conclusion:**

Study revealed that 3 months after bariatric surgery, inflammation persists, and its markers significantly influence postoperative weight loss, as indicated by BMI range. Distinct behaviors of IL-10 and IL-6 in relation to obesity underline the necessity of considering individual cytokine profiles when evaluating bariatric surgery outcomes.

**Graphical Abstract:**

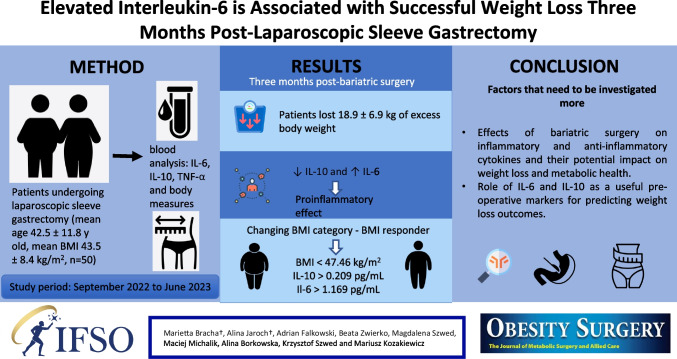

## Introduction

Overweight and obesity are still a pressing global health problem, despite numerous programs and strategies aiming to ease this epidemic. The number of people affected by excess body weight is growing dramatically, and this tendency shows no signs of slowing down [1]. As diet and physical activity become insufficient in obtaining and maintaining weight loss, new strategies are being employed, such as bariatric surgery.

Eligibility criteria for bariatric surgery include BMI greater or equal to 35 kg/m^2^, and even BMI indicating first-degree obesity (30–34.9 kg/m^2^) with comorbidities such as type 2 diabetes, hypertension, obstructive sleep apnea, and dyslipidemia. Laparoscopic sleeve gastrectomy (LSG) is one of the most common types of surgery, well-recognized for its very low risk of complications and proven long-term efficaciousness [2]. A 10-year follow-up revealed a good and sustainable weight loss measured as a percentage of excess body weight loss (%EBWL), remission of type 2 diabetes, dyslipidemia, obstructive sleep apnea, and lower prevalence of Barrett’s esophagus [3]. These beneficial changes resulting from bariatric surgery are associated with strong metabolic change, especially changes in inflammatory biomarkers but also an unpredictability of the inflammation outcomes [4].

Obesity is associated with a chronic inflammatory process of low intensity, defined as metabolic inflammation or “metainflammation” that affects important metabolic tissues such as adipose tissue, liver, skeletal muscle, pancreas, intestines, and hypothalamus [5]. An increased mass of adipose tissue may activate the immune process in the white adipose tissue (WAT), liver, and immune cells [6]. Adipocytes directly release cytokines, but in addition, the immune cells that take up residence in the adipose tissue independently secrete cytokines [7]. Among the most well-studied cytokines concerning obesity are the inflammatory cytokines such as TNF-α (tumor necrosis factor alpha), interleukin-6 (IL-6), and the anti-inflammatory cytokine IL-10 [7].

Considering the link between obesity and inflammation, we aimed to examine the early effects of bariatric surgery (laparoscopic sleeve gastrectomy) on inflammatory and anti-inflammatory cytokines (TNF-α, IL-6, IL-10). We hypothesized that low-grade inflammation is present 3 months after bariatric surgery, and that inflammation marker concentrations have a significant impact on postoperative weight loss.

## Materials and Methods

### Study Participants

This prospective cohort study was conducted from September 2022 till June 2023, according to the guidelines laid down in the Declaration of Helsinki. Study was approved by the local Bioethics Committee. Study participants were adults (age ≥ 18 years old) scheduled for a standard laparoscopic sleeve gastrectomy. Participants were recruited prospectively and consecutively from the bariatric surgical clinic. Written informed consent was obtained from all patients. Exclusion criteria were severe untreated diseases (e.g., psychiatric disorders, addiction to alcohol or drugs, cancer, acute inflammatory diseases) and use of any pharmacological management of obesity, not comorbidities. Measurements were taken 1 week before laparoscopic sleeve gastrectomy and at follow-up 3 months after surgery. All biochemical and anthropometric measurements were taken at both time points.

### Blood Biochemical Analysis

Blood was collected from subjects after 12 h of fasting via the ulnar vein into 6 mL tubes containing EDTA. Within 1 h postcollection, the blood samples were centrifuged for 15 min at 1500 rpm and 4 °C. Subsequently, the plasma was aliquoted into 400-μL portions and transferred into Eppendorf tubes, then stored at − 80 °C until further analysis. Plasma levels of high-sensitivity IL-10 (hsIL-10), high-sensitivity IL-6 (hsIL-6), and high-sensitivity TNF-α (hsTNF-α) were determined using specific enzyme-linked immunosorbent assay (ELISA) kits supplied by Cloud-Clone Corp. (Katy, TX, USA), adhering strictly to the manufacturer’s guidelines. The intra- and inter-assay variabilities were maintained within ± 10/12% for hsIL-10, ± 10/12% for hsIL-6, and ± 10/12% for hsTNF-α with detection sensitivities of less than 0.61 pg/mL, 0.31 pg/mL, and 0.55 pg/mL, respectively. Absorbance readings were taken at a wavelength of 450 nm using a SPECTROstar Nano Microplate Reader (BMG LABTECH, Ortenberg, Germany).

### Anthropometric Measurements

Anthropometric measurements were made in accordance with the principles of nutritional assessment [8, 9]. A total of twenty measurements were obtained: ideal body weight (IBW), typical weight, current weight, height, BMI, overweight (kg), overweight loss (kg), %EBWL, waist, hips waist-to-hip ratio (WHR), waist-to-height ratio (WHtR), body adiposity index (BAI %), body fat (BF%), body fat (BF, kg), resting energy expenditure (REE, kcal), skeletal muscle mass (SMM, kg), skeletal muscle index (SMI, kg/m^2^), fat-free mass (FFM, kg), total body water (TBW, kg). Height was measured with a portable height measure stadiometer (Seca 213). Weight and parameters assessing body composition—body fat (BF), fat-free mass (FFM), skeletal muscle mass (SMM), and skeletal muscle index (SMI)—were measured using the segmental body composition analyzer InBody 570.

Body circumferences—waist and hips—were measured using anthropometric tape with a millimeter scale SECA 201. Measured circumferences allowed to calculate WHR and WHtR. BMI was calculated according to the World Health Organization (WHO) formula. EBWL% was calculated using preoperative weight, weight at follow-up and IBW calculated from Broca’s formula.

### Statistical Analysis

Statistical analyses included parametric and non-parametric tests. The normality of the distribution of the analyzed variable was assessed using the Shapiro–Wilk test. The significance of differences between anthropometric and biochemical variables was calculated using the Student’s *t*-test for paired samples or its non-parametric alternative, the Wilcoxon signed-rank test. Normally distributed continuous variables are described as the mean ± standard deviation (SD), while variables non-normally distributed are expressed as medians (interquartile ranges). Values of body measures were assessed 3 months after surgery in accordance with interleukin concentrations. Based on the calculation of interleukin-6 and interleukin-10 median concentrations, patients were divided into 3 groups: low, medium, and high concentration. The significance of differences was measured using a one-way ANOVA or non-parametric Kruskal–Wallis one-way analysis of variance. After one-way ANOVA, Tukey’s test was used for post hoc analysis. This statistical analysis was performed using STATISTICA 13.3 (version 13.3, StatSoft, Palo Alto, USA).

Next, out of 20 body measures examined, we selected ten continuous variables and added three variables for cytokine concentrations to predict changes in BMI category during the initial 3 months following surgery. As previously, the normal distribution of the data was investigated using Shapiro–Wilk tests. To compare continuous variables between patients who experienced a decrease in BMI category 3 months after surgery (termed “BMI responder”) and those who did not (“BMI non-responder”), we used the independent *t*-test for variables with normal distribution and the Mann–Whitney test for variables without normal distribution.

A decision tree model was developed to predict BMI category change (“responder” or “non-responder”), based on all 13 potential predictors (measured at baseline): weight (kg), BMI (kg/m^2^), overweight (kg), waist circumference (cm), WHR, WHtR, BF%, BF (kg), SMI (kg/m^2^), SMM (kg), TNF-alpha (pg/mL), IL-10 (pg/mL), IL-6 (pg/mL). The model was tested through leave-one-out cross-validation. In brief, given a dataset with n observations, this technique partitions the data into two subsets for each iteration of the model evaluation process. Specifically, for each iteration, one observation is designated as the test set, while the remaining n − 1 observations form the training set. The model is subsequently trained on these n − 1 observations and then tested on the single reserved observation. This procedure is iterated n times, ensuring each of the n observations serves as the test set exactly once. After each iteration, the prediction error for the solitary observation used as the test set is documented. The overall prediction error is then calculated as the mean of the errors from the n models constructed during the cross-validation process.

The decision tree algorithm was used with a Gini impurity measure, a maximum tree depth of 3, a minimum of 2 cases in parent nodes, and at least 1 case in child nodes. To prevent overfitting, the algorithm underwent cost-complexity pruning with the parameter alpha set to 0.01. The statistical and machine learning methodology used in this study are comprehensively explained in [10].

The model’s performance was rated using well-established metrics such as accuracy, recall, precision, F1, and the area under the receiver operating characteristic curve (AUC) and the corresponding *p*-value calculated using the Mann–Whitney U test. These performance indicators were calculated as follows:

- Accuracy = (TP + TN) / (TP + FP + FN + TN).
- Recall = TP / (TP + FN).
- Precision = TP / (TP + FP).
- F1 Score = 2 / ((1 / recall) + (1 / precision)).

Here, TP represents the count of true positives, TN denotes true negatives, FP is the false positives, and FN indicates false negatives. The development and analysis of prediction models were conducted with a custom Python script, incorporating the sklearn and SciPy libraries.

## Results

Fifty-four patients scheduled for sleeve gastrectomy were assessed for eligibility 1 week before surgery. Among them, four met the exclusion criteria, and eight chose not to participate in the study. Of the 42 participants enrolled, two declined to take part in the follow-up due to non-medical reasons. Consequently, 40 of them had biochemical profiling performed (mean age 41.6 ± 11.4 years old, 30 women) (Table 1).

**Table 1:** Anthropometric and biochemical characteristics

	Baseline	Follow-up	*p*
Weight, kg	123.3 ± 27.1	104.3 ± 23.7	< 0.0001
BMI, kg/m^2^*	41.52 (35.50–50.80)	35.68 (29.50–41.34)	< 0.0001
Overweight, kg*	52.6 (34.5–75.0)	32.5 (19.8–49.6)	< 0.0001
Waist, cm	122.1 ± 16.2	106.7 ± 15.8	< 0.0001
Hips, cm	133.7 ± 17.0	122.3 ± 16.9	< 0.0001
WHR	0.92 ± 0.09	0.88 ± 0.10	< 0.0001
WHtR	0.72 ± 0.096	0.63 ± 0.097	< 0.0001
BF, kg	60.7 ± 19.4	45.8 ± 18.2	< 0.0001
SMM, kg	35.1 ± 6.9	32.2 ± 6.1	< 0.0001
SMI, kg/m^2^*	8.9 (8.2–10.3)	8.5 (7.4–9.7)	< 0.0001
IL-10, pg/mL*	0.478 (0.220–2.255)	0.051 (0.036–0.057)	< 0.0001
IL-6, pg/mL*	1.144 (0.986–1.345)	5.201 (3.534–8.295)	< 0.0001
TNF-α, pg/mL*	2.267 (2.187–2.400)	2.290 (2.185–2.345)	0.7063

*BMI* Body Mass Index, *WHR* waist-to-hip ratio, *WHtR* waist-to-height ratio, *BF* body fat, *SMM* skeletal muscle mass, *SMI* skeletal muscle index, *TNF-α* tumor necrosis factor alpha, *IL-10* interleukin-10, *IL-6* interleukin-6

*Median and interquartile values shown as data did not have a normal distribution

All anthropometric and biochemical parameters, except for TNF-α, differed significantly 3 months after bariatric surgery (Table 1). Patients lost on average 18.9 ± 6.9 kg of excess body weight (%EBWL 37.6 ± 15.5%), and their BMI values began to indicate second-degree obesity (35.68 kg/m^2^). A significant decrease was observed for anti-inflammatory interleukin-10 concentration, simultaneously with a significant increase in pro-inflammatory interleukin-6.

Interleukin concentrations were compared with anthropometric measurements (BMI, overweight, overweight loss, waist, hips, WHR, WHtR, body fat, SMM, SMI) at follow-up. For this comparison, IL-10 and IL-6 follow-up concentrations were sorted into three groups: low, medium, and high concentration (Table 2).

**Table 2:** IL-10 and IL-6 concentrations grouping (follow-up measurements)

	Follow-up median concentration	Min–max
IL-10, pg/mL	0.051 (0.036–0.057)	
1 low (*n* = 16)	0.031 (0.029–0.040)	0.025–0.040
2 medium (*n* = 19)	0.053 (0.051–0.058)	0.046–0.066
3 high (*n* = 5)	0.100 (0.100–0.103)	0.096–0.113
IL-6, pg/mL	5.200 (3.534–8.295)	
1 low (*n* = 12)	2.971 (2.305–3.408)	1.608–3.818
2 medium (*n* = 17)	5.202 (4.574–6.039)	4.137–7.611
3 high (*n* = 11)	15.159 (8.374–50.720)	8.220–63.653

*n* number of patients

**Fig. 1:**
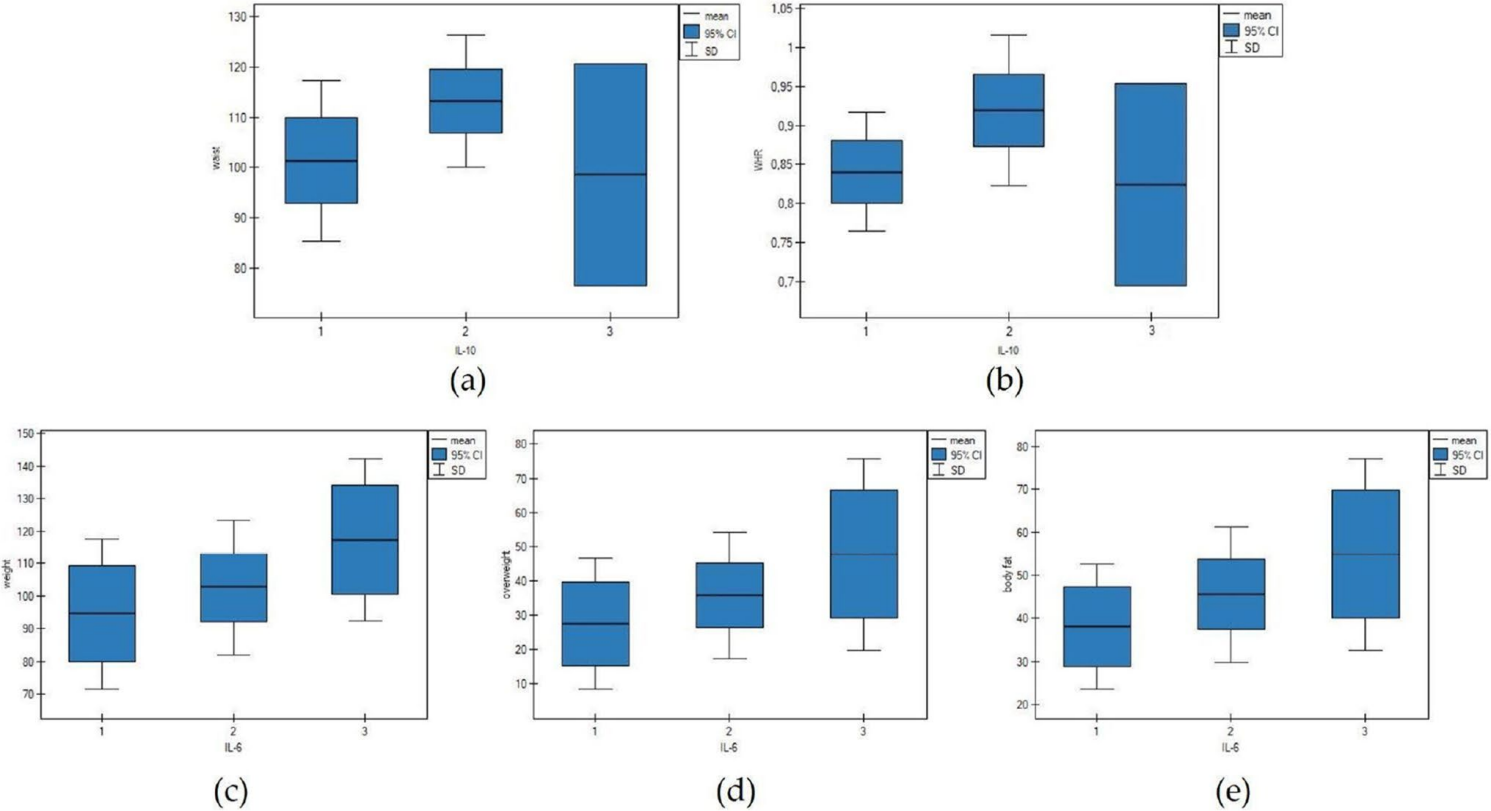
Box plots for IL-10 and IL-6 concentrations (pg/mL) (1, low concentration; 2, moderate concentration; 3, high concentration) and anthropometric parameters: **a** IL-10 and waist circumference (cm); **b** IL-10 and waist-to-hip ratio (WHR); **c** IL-6 and weight (kg); **d** IL-6 and overweight (kg); **e** IL-6 and body fat (kg)

Analyzing IL-10 follow-up concentrations’ significant differences were found for waist circumference (*p* = 0.0389) and WHR (*p* = 0.0199). Further post hoc analysis showed no significant difference for waist, while WHR value differed significantly between groups with low and moderate IL-10 concentration (*p* = 0.0343). It was noticeable in box plot analysis that low and high IL-10 concentrations were associated with lower values of anthropometric parameters, whereas the relationship between IL-6 follow-up concentrations and anthropometric values was linear—with increasing IL-6 values of anthropometric parameters also increased (Fig. 1).

Further analysis was aimed at investigating whether there is a parameter or group of parameters which would be predictive of a substantial decline in body weight. Thus, continuous variables were compared between patients who experienced a decrease in BMI threshold (e.g., from second-degree obesity to first-degree) 3 months after surgery (termed “BMI responder”) and those who did not (“BMI non-responder”). A decision tree model was developed to predict a decrease in BMI threshold, based on finally ten potential predictors at baseline values, and a significant difference was found for several variables (Table 3).

**Table 3:** Differences in examined parameters according to a decrease in BMI threshold

	BMI responder Shapiro–Wilk; *p*	BMI non-responder Shapiro–Wilk; *p*	*t*-test; *p*	Mann–Whitney *U* test; *p*
Weight (kg)	0.135	0.135	< 0.0001	< 0.0001
BMI (kg/m^2^)	0.247	0.247	< 0.0001	< 0.0001
Overweight (kg)	0.483	0.483	< 0.0001	< 0.0001
Waist (cm)	0.073	0.073	< 0.0001	< 0.0001
WHR	0.405	0.405	0.937	0.817
WHtR	0.446	0.446	< 0.0001	< 0.0001
BF (%)	0.001	0.001	< 0.0001	< 0.0001
TNF-α (pg/mL)	< 0.0001	< 0.0001	0.581	0.225
IL-10 (pg/mL)	< 0.0001	< 0.0001	0.616	1
IL-6 (pg/mL)	0.611	0.611	0.304	0.299

*BMI* Body Mass Index, *WHR* waist-to-hip ratio, *WHtR* waist-to-height ratio, *BF* body fat, *TNF-α* tumor necrosis factor alpha, *IL-10* interleukin-10, *IL-6* interleukin-6

**Fig. 2:**
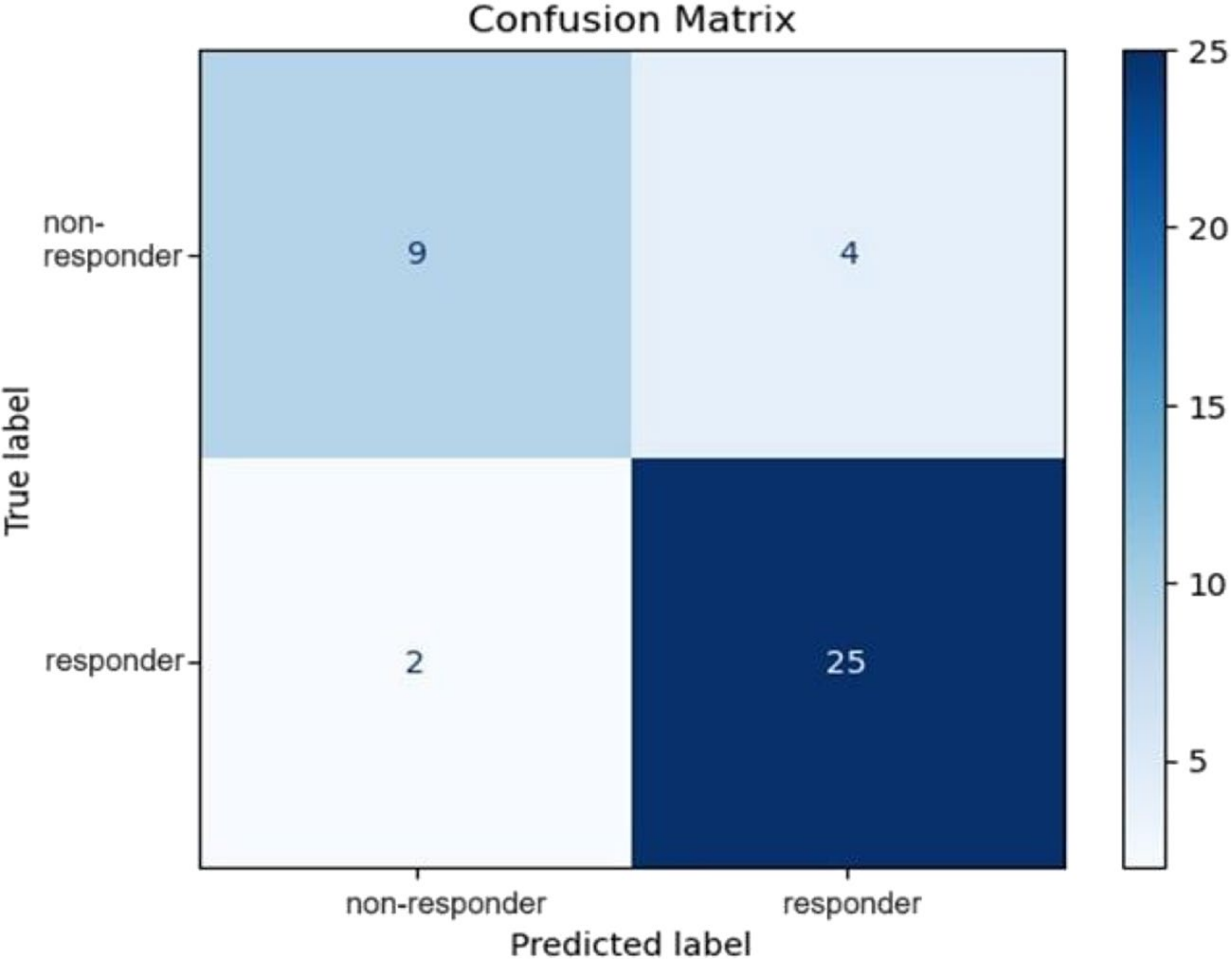
Confusion matrix for BMI prediction

In the leave-one-out cross-validation procedure, the decision tree model achieved an AUC of 81%, with a *p*-value < 0.001. The confusion matrix showed that the model correctly classified change status in 25 out of 27 participants in the BMI responder group and 9 out of 13 participants in the BMI non-responder group (Fig. 2). This resulted in an accuracy of 85%, a recall of 93%, a precision of 86%, and an F1 score of 89%.

**Fig. 3:**
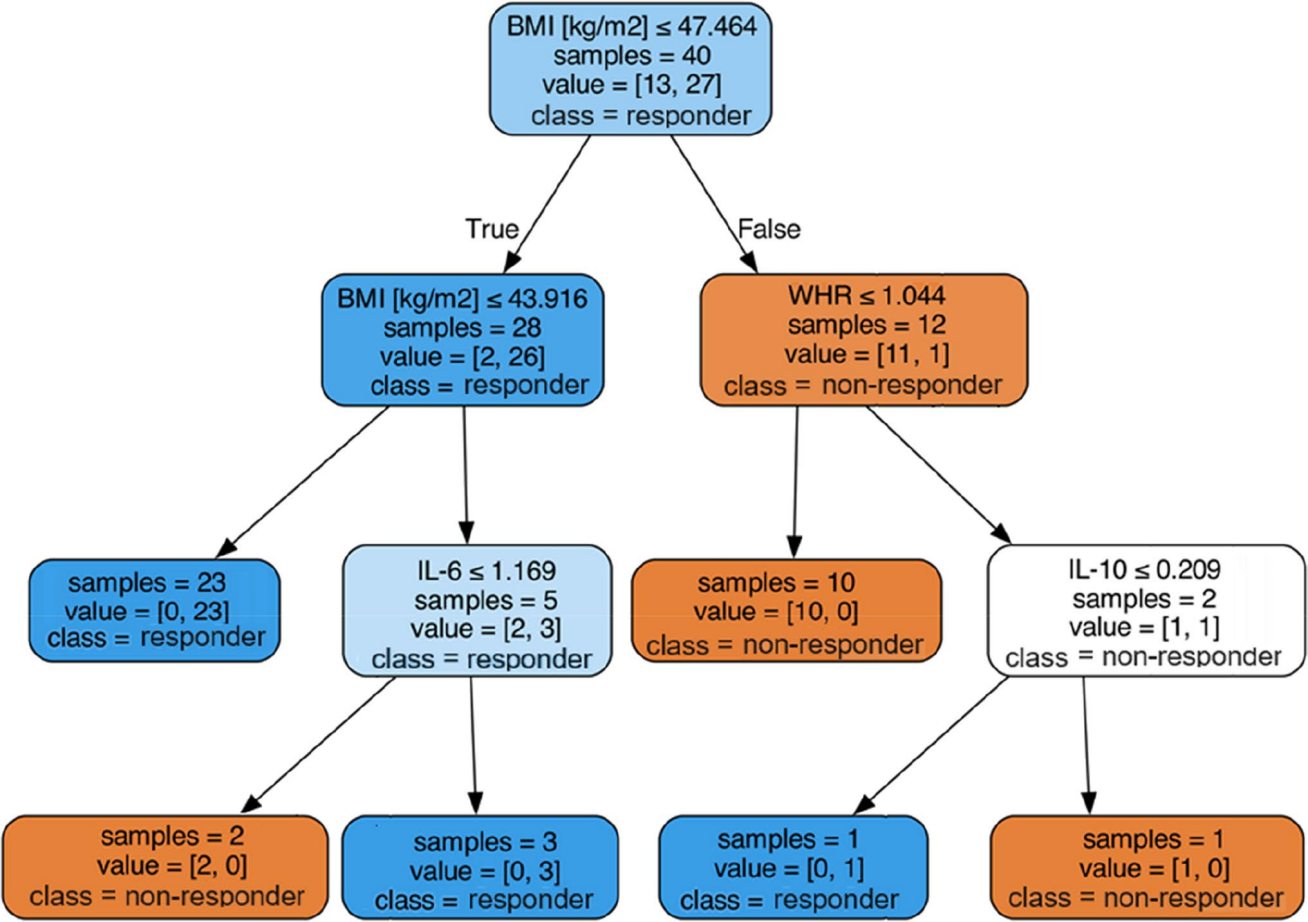
Decision tree

The decision tree was created to investigate predictors of BMI change 3 months postsurgery. It showed that participants with a baseline BMI of at least 47.46 kg/m^2^ had a minimal chance of changing their BMI category, with only 1 out of 12 achieving this. A low IL-10 baseline level (less than or equal to 0.209 pg/mL) could assist in this scenario. On the contrary, participants with a baseline BMI less than 47.46 kg/m^2^ had a significant chance of changing their BMI. However, low IL-6 baseline levels (less than or equal to 1.169 pg/mL) could be a risk factor for not achieving weight loss 3 months after surgery (Fig. 3).

## Conclusion

In this study, we have demonstrated that 3 months after bariatric surgery the level of anti-inflammatory IL-10 significantly decreased, while pro-inflammatory IL-6 was significantly higher. This pro-inflammatory effect is a result of weight loss, especially among patients with a BMI < 47.46 kg/m^2^ at baseline. Moreover, we found that patients with a baseline BMI ≥ 47.46 kg/m^2^ were considerably less prone to lose body weight in terms of changing their BMI category.

Significant postoperative change in interleukin-10 levels coupled with an increase in interleukin-6, and suggests an exacerbation of the inflammatory state, particularly with an observed increase in TNF-alpha, although not statistically significant. This finding aligns with other studies indicating that while IL-6 and TNF-alpha are closely linked to inflammation, their changes may not always correlate with statistical significance in clinical outcomes [6]. It is widely known that chronic inflammation is inextricably linked with adipose tissue hypertrophy. This process is expressed through an increase in pro-inflammatory TNF-alpha and IL-6 concentrations, simultaneously with an IL-10 decrease reflecting an impaired regulatory mechanism against inflammation [11]. In the presented study, patients experienced a significant, rapid weight loss; at only 3 months after surgery, they had already lost a mean of almost 19 kg, equating to 37.6% loss of excess body weight. In line with other authors, body circumferences (waist, hips) and body composition compartments (BF, SMM) also significantly decreased [12]. Inflammatory factors (IL-6, TNF-α, CRP) are hypothesized to decrease after bariatric surgeries, but typically studies focus on follow-up measurements made at six or 12 months after bariatric surgery [6]. In our study, we decided to examine our patients early on postsurgery, and discovered that IL-6 follow-up concentration was significantly higher than baseline. This may be explained by the fact that postsurgical recovery and/or weight loss can induce an increase in free fatty acid release, which triggers inflammation [13, 14]. Many studies showed that bariatric surgery reduces circulating levels of pro-inflammatory marker IL-6, but not typically before 6 months [15–19].

Our study showed a non-significant increase (*p* = 0.7063) in TNF-alpha serum level 3 months after bariatric surgery, and this outcome is in line with the findings of other researchers [20–22]. Kelly et al. reported a slight reduction in TNF-α in the long-term observation, while for short-term results TNF-α level was increased; in either case, no statistically significant difference was found [23]. This pattern could be attributed to the multifaceted roles of TNF-alpha in inflammation and its regulation, which may not always be directly measurable in short-term or small-scale studies. Additional insights from Lira et al. suggest that reductions in visceral fat significantly correlate with decreases in pro-inflammatory markers like IL-6 and TNF-alpha, indicating that changes in body composition can influence cytokine profiles, which may explain variations in TNF-alpha significance across studies [24]. According to a meta-analysis, at least 12 months are required before a consistent decrease in TNF-α is noticed [25].

After grouping IL-6 follow-up levels into three concentrations (low, medium, high), we found that with rising IL-6 concentrations, the values of anthropometric measurements (body weight, overweight, and body fat mass) also increased 3 months after LSG; however, these results were not statistically significant. Adipose tissue is an important endocrine organ secreting several inflammatory markers, and a number of adipokines, such as adiponectin, leptin, and resistin. In the state of obesity, the pro-inflammatory adipokines derived from adipose tissue are overexpressed, and among which, increased production and secretion of inflammatory mediator IL-6 is marked [26]. Further, these inflammatory markers stimulate oxidative stress and cause inflammation [27]. The lack of significant differences between IL-6 follow-up levels and anthropometric parameters might indicate that while IL-6 is elevated in obese states, it may not directly correlate with changes in body dimensions over short-term interventions. This is supported by the broader literature which suggests that IL-6 levels are more reflective of acute inflammatory responses rather than changes in adiposity [28].

The same grouping was performed for IL-10 follow-up concentrations. IL-10 is a major anti-inflammatory and immunoregulatory cytokine that inhibits inflammatory, and cell-mediated immune responses [29]. Significant differences were observed in the levels of IL-10 and anthropometric measurements such as waist circumference and WHR at follow-up time point. It can be generalized that with increasing IL-10 concentrations, body measures also increased. We observed that in the group with high IL-10 concentration, anthropometric parameters decreased; however, there were only five patients in this group. This finding aligns with previous research showing that IL-10, an anti-inflammatory cytokine, has a complex interplay with metabolic factors in obese populations. For example, Esposito et al. noted that low baseline IL-10 levels correlate with obesity and metabolic syndrome, suggesting that improving IL-10 levels could ameliorate some metabolic disturbances in obesity [30].

Regarding the impact of high BMI and cytokine levels such as IL-10 and IL-6 on weight loss effectiveness is rooted in a deep understanding of their roles in weight regulation and inflammatory response within the body. High BMI often poses significant challenges in achieving substantial and lasting weight reduction through conventional weight loss methods, such as diet and exercise, due to complex metabolic and psychological barriers [31]. IL-10, an anti-inflammatory cytokine, plays a protective role against chronic inflammation associated with obesity. Lower levels of IL-10 may contribute to persistent inflammation, complicating weight loss efforts. Research shows that patients with lower IL-10 levels experience higher inflammation, negatively impacting weight loss [32]. IL-6, on the other hand, has both pro-inflammatory and anti-inflammatory effects, depending on the context.

This study presents potential limitations. Firstly, the inflammation panel included interleukins and TNF-alpha but did not measure C-reactive protein (CRP). We opted to concentrate on the role of cytokines in the inflammatory response following bariatric surgery. Given that IL-6 significantly influences the hepatocytic secretion of acute-phase proteins, including CRP, we can reasonably infer that CRP levels would also be elevated. We decided to use a decrease in BMI category within the decision tree model because of the very limited literature on a specific %EBWL threshold that a patient should achieve as early as 3 months postbariatric surgery. Each BMI category encompasses a specific range, and transitioning to a different BMI category is more feasible when the initial BMI is near the threshold of that range. However, the first 3 months following surgery typically coincide with a significant reduction in body mass, thereby facilitating the shift to a different BMI category. Another limitation is the relatively small sample size, which was constrained by the exclusion criteria, number of surgeries scheduled, and focusing on assessing inflammation associated with a single type of bariatric surgery procedure. Lastly, as previously stated, there were only five patients in the group with high IL-10 follow-up levels. This unique group, categorized according to IL-10 levels, cannot be subdivided differently; a larger sample size is necessary to ensure a more balanced patient distribution.

Our study revealed that 3 months after bariatric surgery, inflammation persists, and its markers significantly influence postoperative weight loss, as indicated by BMI range. Interactions among cytokines within inflammatory pathways are complex, especially in obesity-related inflammation, where maintaining a balance between pro-inflammatory and anti-inflammatory cytokines is crucial. The distinct behaviors of IL-10 and IL-6 in relation to obesity underline the necessity of considering individual cytokine profiles when evaluating inflammation and obesity interventions. It highlights the potential for targeted therapies that specifically modulate cytokine activity to improve obesity-related outcomes. Such insights are crucial for developing more effective treatments for obesity, which address both metabolic and inflammatory components of the disease.

## Data Availability

Data available in the Research Data Repository of Nicolaus Copernicus University in Toruń, Collegium Medicum in Bydgoszcz, Poland.
