# Correction: Elevated Interleukin-6 Is Associated with Successful Weight Loss 3 Months Postlaparoscopic Sleeve Gastrectomy

**DOI:** 10.1007/s11695-025-08260-2

**Published:** 2025-09-15

**Authors:** Marietta Bracha, Alina Jaroch, Adrian Falkowski, Beata Zwierko, Magdalena Szwed, Maciej Michalik, Alina Borkowska, Krzysztof Szwed, Mariusz Kozakiewicz

**Affiliations:** 1 Department of Geriatrics, Division of Biochemistry and Biogerontology, Faculty of Health Sciences, Collegium Medicum in Bydgoszcz, Nicolaus Copernicus University in Torun, Bydgoszcz, 85626 Poland; 2 Department of Clinical Neuropsychology, Faculty of Health Sciences, Collegium Medicum in Bydgoszcz, Nicolaus Copernicus University in Torun, Bydgoszcz, 85094 Poland; 3 Department of Probability Theory and Stochastic Analysis, Faculty of Mathematics and Computer Science, Nicolaus Copernicus University in Torun, Torun, 87100 Poland; 4 Department of General and Minimally Invasive Surgery, Faculty of Medicine, Collegium Medicum in Bydgoszcz, Nicolaus Copernicus University in Torun, Bydgoszcz, 85168 Poland; 5 The Mazovian Academy in Plock, Plock, 09402 Poland

**Correction: Obesity Surgery (2024) 34:3824-3832**

10.1007/s11695-024-07468-y

A funding source was missing in the published article: This work was supported by the National Science Center (Poland), MINIATURA 7 grant (research activity number 2023/07/X/NZ5/00422).

